# Comparison of antifungal efficacy of ethanolic extracts of *Woodfordia fruticosa* leaf and *Punica granatum* peel in uncontrolled diabetic patients wearing removable dentures: A randomized controlled clinical trial

**DOI:** 10.18502/cmm.6.3.3983

**Published:** 2020-09

**Authors:** Bhavana Sujanamulk, Salavadhi Shyam Sunder, Babita Ratnakar Pawar, Chintamaneni Rajalakshmi, Kotya Naik Maloth

**Affiliations:** 1 Department of Oral Medicine and Radiology, Drs Sudha and Nageswararao Siddhartha Institute of Dental Sciences, Gannavaram Mandal, Krishna District, Andhrapradesh, India; 2 Department of Periodontics, Chhattisgarh Dental College and Research Institute, Rajnandgaon, Chhattisgarh, India; 3 Department of Oral Medicine and Radiology, Mamata Dental College, Khammam, Telangana, India

**Keywords:** Antifungal agents, Glycosides, Oral candidiasis, *Punica granatum*, *Woodfordia fruticosa*

## Abstract

**Background and Purpose:**

The search for the development of a suitable novel antimicrobial agent for fungal diseases continues to be a key problem in the current clinical field. The present investigation was aimed to determine the antifungal effect of the ethanolic crude extracts of *Woodfordia fruticosa* leaf (Wfl) and Punica granatum peel (Pgp) in uncontrolled diabetic patients wearing removable dentures.

**Materials and Methods:**

The ethanolic extracts of both plants were prepared using the soxhlet extraction method, and the obtained metabolites were confirmed by thin- layer chromatography. After the preparation of the mouthwash, a total of 100 subjects were randomly divided into two groups. Each subject was given physiological saline at the baseline. Group I was provided with *P. granatum* mouthwash, while Group II was given *W. fruticosa* mouthwash. Following the administration of the mouthwash, the patients were requested to rinse the mouthwash using the oral rinse technique twice daily 5 ml/rinse for 30 sec. Subsequently, colony-forming units (CFU) were evaluated in the participants. Post-therapeutic samples were collected 1 h and 1 week after the mouthwash use.

**Results:**

The mean reduction of CFU was calculated at the baseline, as well as 1 h and 1 week after using mouthwash. The results indicated a drastic reduction in CFU 1 h and 1 week after the application of Wfl mouthwash.

**Conclusion:**

The obtained data revealed that Wfl had potential anticandidal activity against *Candida* yeast cells, probably owing to its bioactive compounds like glycosides. Therefore, this agent can be used effectively as a natural remedy for the treatment of oral candidiasis. However, the exact mechanism of action of this plant needs to be elucidated.

## Introduction

Oral candidiasis is an opportunistic fungal infection that is more prevalent in patients with xerostomia or immunosuppressive conditions (e.g., HIV infection), denture wearers, and those on anticancer chemotherapy or high carbohydrate diet. Moreover, this infection is considered to be a hallmark of systemic diseases, such as diabetes mellitus [ 1 ]. *Candida* species, namely *C. albicans*, *C. glabrata*, and *C. tropicalis*, account for 80% of fungal infections in the oral cavity. *Candida* species have the isolation rates of 20-75% and 50-65% from the oral cavity in the general population and individuals wearing removable dentures, respectively [ 2 , 3 ].

The widespread use of antifungal agents, particularly azole drugs, has caused a considerable increase in clinical resistance and failure to respond in the current scenario of fungal diseases [ 4 ]. This has implicated the utmost need for replacing chemical drugs with herbal medicines, which date back centuries ago. There are drawbacks to the conventional antifungal arsenal due to the high toxicity of the compounds, cost factors, drug interactions, and more importantly antimicrobial resistance. Therefore, there is a substantial need to explore a novel antifungal agent that enhances the bioavailability in order to improve the antifungal spectrum and combat resistance [ 5 ]. The plants of Lythraceae, also called the loosestrife family, have either wild or cultivated species and have gained medicinal importance due to their numerous pharmacological activities [ 6 ]. With an aim to discover a novel antifungal agent, the present study was conducted to investigate the antifungal activity of two plants from the same family, namely **Punica* granatum* (Pg) and *Woodfordia fruticosa* (Wf), for uncontrolled diabetic patients wearing removable dentures.

The Wf, also called fire flame bush or Dhawai, contains various natural compounds, such as tannins, phenols, terpenoids, steroids, and carbohydrates [ 7 ]. There is evidence on the in vitro antimicrobial effect of Wf leaves and flowers; however, these effects have not been examined in any in vivo studies till date. The results of multiple studies, such as those conducted by Parekh *et al*. (2007) [ 8 ], Kumaraswamy *et al*. (2008) [ 9 ], Chougale *et al*. (2009) [ 10 ], Kaur *et al*. (2010) [ 11 ], and Kharate *et al*. (2018) [ 12 ], have confirmed the inhibitory effect of Wf against various bacterial strains.

The Pg, also called pomegranate, is a fruit distributed worldwide. It has innumerable bioconstituents, such as flavonoids, polyphenols, terpenoids, alkaloids, tannins, ellagic acid, and gallic acid [ 13 ]. The findings of various studies, such as those performed by Vasconcelos *et al*. (2003, 2006) [ 14 ], Duman *et al*. (2009) [ 15 ], Anibal *et al*. (2010) [ 13 ], Ferrazzano et al. (2017) [ 16 ], and Bassiri-Jahromi *et al*. (2015, 2018) [ 17 ], are suggestive of the potent antifungal efficacy of Pg and its role in the inhibition of the growth of various *Candida* species. The overview of the literature on these two plants indicates the scarcity of the studies investigating the antifungal effect (especially with regard to Wf) against *Candida* species. Regarding this, the present study was conducted to specifically examine the antifungal activity of the ethanolic extracts of these two plants in uncontrolled diabetic patients wearing removable dentures. It was also aimed to identify and isolate the active substance in both plants.

## Materials and Methods

The selected plants, namely Wf and Pg, were collected from the SM Heena Industries in India in June 2019. The chosen plants were taxonomically authenticated and identified at the Deccan branch, Botanical Survey of India Hyderabad, with the herbarium voucher No. D-95.

**Extraction of secondary metabolites and biochemical test for *Woodfordia fruticosa* leaf and **Punica* granatum* peel**

The dried Wf leaves (Wfl) and Pg peel (Pgp; 1500 gm) were put in a mechanical grinder. The obtained coarse powder was then subjected to extraction with ethanol using a soxhlet extractor. Finally, the obtained extract was dried in a vacuum using a rotary evaporator. The yield percentage was calculated using the following formula [ 18 ]:

Extract yield%=R/S×100

where R is the weight of the extracted plant residues and S is the weight of the raw plant sample.

For phytochemical analysis, the extracts were dissolved in ethanol (1 mg/ml). Phytochemical screening was performed, and biochemical tests were conducted. Some of the secondary metabolites found in Wfl were glycosides and carbohydrates, and those in Pgp were flavonoids, steroids, and terpenoids.

To confirm the presence of these phytoconstituents, high-performance thin-layer chromatography (TLC) was carried out. In doing so, the mobile phase of ethanol: isopropyl alcohol: triethylamine was taken at the ratio of 6:5:3:5:1.0, and the stationary phase of silica gel was used. The spraying reagents, like glacial acetic acid and concentrated sulfuric acid, were used to visualize the number of spots. Based on color and retention factor (Rf) value, the plates were visualized at visible light with the UVs of 254 and 366 nm, respectively. The Rf value was calculated using the following formula (Kumar S *et al*., 2013) [ 19 ]:

Rf=Distance travelled by component Distance travelled by solvent

The identified metabolites were then separated by means of TLC.

**Preparation of mouthwash**

To prepare the mouthwash, 0.8 gm of each plant extract was superadded with 10 ml glycerol, 0.2 gm L- menthol, 0.05 gm peppermint oil, and 0.2 gm citric acid. Subsequently, sufficient water was added to make a total volume of 100 ml. The mouthwash was formulated according to the procedure adopted by Sritrairat *et al*. (2011) [ 20 ].

**Study population**

The present study was conducted at the Department of Oral Medicine and Radiology between 2019 and 2020. The study procedures were in accordance with the CONSORT and Declaration of Helsinki (revised in 2013) guidelines. The study protocol was approved by the Institutional Ethics Committee of the Central Drugs Standard Control Organisation (No. ECR/804/Inst/AP/2016). Informed consent was obtained from all the subjects willing to participate in the research. The present study was a continuation of our previous research published in the Journal of Clinical and Diagnostic Research in 2016 with the Clinical Trial Registry number of REF/2016/03/11053.

A total of 100 subjects who were diabetic and wore denture, either removable or fixed types, were randomly selected by a simple randomization method using a coin toss. The participants were evaluated for blood sugar levels by glycosylated hemoglobin assay, and the test results were standardized according to the American Diabetes Association. Those with a hemoglobin A1C level of > 7 were considered poor diabetic and included in the study. Some of the patients had such clinical symptoms as the burning and painful sensation of oral cavity or change of taste; however, most of them were asymptomatic. The inclusion criteria were: 1) pregnancy or lactation, 2) allergy to prepared mouthwash, 3) diagnosis of other systemic diseases, and 4) use of herbal products or form of mouthwash in the last 2 months.

**Study groups and procedure for the estimation of antifungal activity**

A total of 100 subjects were investigated in this double-blinded study in two groups based on the coin toss (n=100, age: 35-65 years, both women and men). Group I (n=50) were provided with Pgp mouthwash, while Group II (n=50) was given Wfl mouthwash. The participants, as well as the physician, were blinded to the allocated product. Following the administration of the mouthwash, the patients were requested to rinse the mouthwash using the oral rinse technique. The samples were collected at three time points. In this regard, at the baseline, each subject was asked to rinse his/her mouth using physiological saline; subsequently, the swish (i.e., amount of mouthwash mix collected from patients' mouth after using mouthwash) was collected in sterile containers, which was then subjected to CFU estimation.

Later, the subjects were randomly allotted to use either of the mouthwash, and the post-therapeutic samples were collected after 1 h and 1 week. The subjects were advised to use the given mouthwash twice a day (5 ml/rinse for 30 sec). They were also instructed to write about their compliance in a diary on a daily basis to obtain a home assessment of the treatment. The participants’ subjective satisfaction with the mouthwash taste and smell was assessed using a 9-point hedonic scale [ 21 ]. In this regard, positive numbers were interpreted as moderate to strong satisfaction, whereas negative numbers were representative of weak satisfaction.

The burning sensation was assessed using the Visual Analog Scale [ 22 ]. In addition, allergy to the prescribed mouthwash was evaluated at different time points. After the collection of samples in sterile containers, a serial dilution technique (3 dilutions for each sample) was performed where 0.2 ml of each sample was diluted in 9.8 ml of physiological saline. The third dilution was used for the determination of the total microbial count. The CFU was estimated using the following formula:

CFU/mL=1,000×number of colonies/3

**Figure 1:**
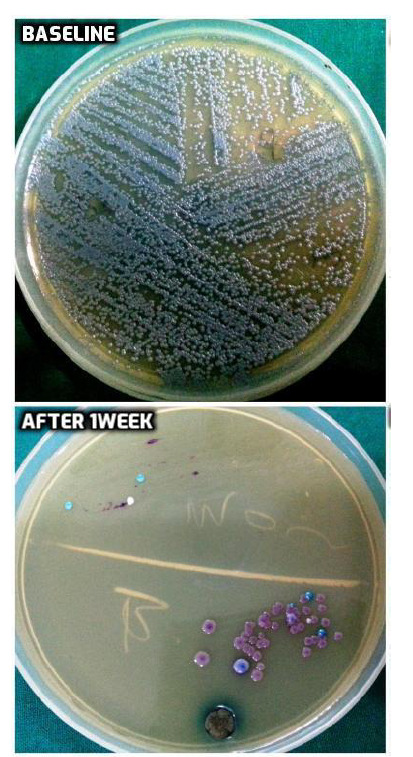
Colony forming units in HiChrome agar at the baseline and 1-week time after using *Woodfordia* leaf mouthwash

The identification of *Candida* species was accomplished using HiChrome *Candida* differential agar (HiMedia Lab, Mumbai, India). In this medium, the observation of green, metallic blue, and cream or white colors were regarded to denote *C. albicans*, *C. tropicalis*, and *C. glabrata*, respectively (Figure 1).

**Statistical analysis**

The data were analyzed in SPSS software (version 21.0) for windows. The data were expressed as mean±standard deviation. In addition, the confidence interval was calculated using inferential statistics to find the uncertainties associated with the sampling method. A p-value of ≤ 0.05 was considered statistically significant, and a p-value of ≤ 0.01 was regarded as highly significant.

## Results

**Figure 2:**
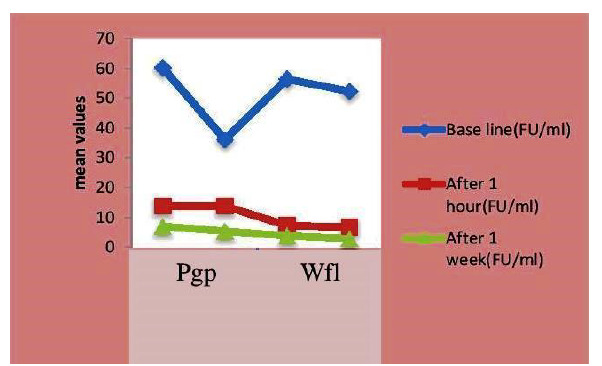
Line graph representing mean reduction of colony forming units per millimeter at different time points in both groups

The results were indicative of a decrease in the mean CFU count in both groups at different time points. This mean value showed a drastic reduction in the Wfl group both 1 h and 1 week after the intervention. Accordingly, Wfl mouthwash was more effective in reducing CFU count at all-time points. This result can be attributed to the excellent antifungal activity of Wfl (Figure 2). Given the higher antifungal efficacy of Wfl mouthwash, a spectral analysis was performed on this product in order to explore the exact phytoconstituent responsible for its antifungal effect.

**Spectral analysis of *Woodfordia fruticosa* leaf**

The interpretation of the spectral data was based on physicochemical properties and their comparison with the data presented in the literature. The functional groups, number of protons and carbons in the structure, and molecular weight of the compound were recorded by the infrared spectra, proton nuclear magnetic resonance spectroscopy, carbon-13 nuclear magnetic resonance, and mass spectrometry, respectively. The spectral analysis revealed glycosides and carbohydrates as the bioconstituents of Wfl. The details are described in The details are described in Table 1.

**Table 1:** Quantitative analysis of *Woodfordia fruticosa* leaf showing chemical characterization

3rPosition	C atom	δ ^13^C (ppm)	δ ^1^H (ppm)
1	C	145.11	-
2	CH	94.40	6.25
3	C	153.71	-
4	CH	95.51	6.24
5	C	157.16	-
6	CH	92.25	6.13
1	OCH_3_	57.41	3.96-3.90
3	OCH_3_	56.83	3.746-3.70
1^1^	CH	100.83	4.97(Glu-H)
2^1^	CH-OH	78.23	3.29 (Glu-H)
3^1^	CH-OH	77.51	3.41(Glu-H)
4^1^	CH-OH	74.71	3.38(Glu-H)
5^1^	CH-OH	77.26	3.27(Glu-H)
6a^1^	CH-OH	69.76	3.28(Glu-H)
6b^1^	CH-OH	69.76	3.26(Glu-H)

C: Carbon atom, δ: delta scale showing chemical shifts, ^13^C: carbon- 13, ppm: parts per million,^1^H: hydrogen-1, CH-methane, CH-OH - Hydroxy methyl group, OCH_3_- Oxygen ,methyl group- Methoxygroup, Glu-H: glucosyl ions

## Discussion

In recent times, the rise in the incidence of fungal infections and clinical resistance to conventional antifungal therapy has implicated an imperative need for the introduction of novel, preventive, and therapeutic approaches for the treatment of oral candidiasis [ 23 ]. Appropriate target identification and rational drug design technologies using the small molecules of natural products can accelerate the development of antifungal agents [ 24 ]. There has been much emphasis on the in vitro antimicrobial activity of Wf. Moreover, the results of several studies have been confirmative of the antifungal activity of *Punica*. However, to the best of our knowledge, the antifungal activity of the ethanolic extracts of these two plants has not yet been discussed in patients with uncontrolled diabetes.

Endocrine disorders, such as diabetes mellitus, can be a predisposing factor for oral candidiasis. The review of the literature in such databases as the Embase, Medline, Science citation index, NIH public access, PubMed, and Cochrane database of systematic reviews, revealed no relevant information on this type of clinical trial. Hence, our research particularly targeted the antifungal activity of the ethanolic extract of these two plants in patients with uncontrolled diabetes. Before the implementation of the present in vivo trial, the toxic study parameters were considered based on a study performed by Palande *et al*. [ 25 ] addressing the acute oral toxicity of Wfl in Wistar rats and proving its safety up to a dose of 2,000 mg/kg.

Likewise, Patel *et al*. [ 26 ] examined the acute toxicity of pomegranate peel extract (PPE) and revealed that it could be safely administered up to a dose of 5,000 mg/kg. Hence, in the present research, 1,500 mg of each plant was used, which was within the determined limit. The ethanolic extracts were preferred in the current study due to their capacity to penetrate the cellular membrane and facilitate the extraction of the intracellular ingredients from the plant, resulting in enhanced efficacy against *Candida* isolates. Moreover, ethanol is considered to be the best solvent for the extraction of the majority of phytoconstituents [ 27 ].

In the present study, the ethanolic extract of Wfl mouthwash showed a superior antifungal activity with respectively 50% and 85% decrease in viable colonies 1 h and 1 week after the intervention, compared to Pgp. Similar to our study, Dubey *et al*. reported effective antimicrobial activity for the ethanolic extracts of Wf, and their findings clearly supported our study [ 28 ]. Kharate *et al*. also determined the antibacterial activity of the methanolic, hexane, chloroform, and acetone extracts of Wf, where methanolic extract showed superior antimicrobial activity [ 12 ]. Similarly, Dabur *et al*. evaluated the antibacterial activity of Wf against various bacterial strains and *C. albicans* using different solvents and observed efficient activity against the mentioned microorganisms, which is consistent with our research findings [ 29 ].

In another study performed by Sahu *et al*. [ 7 ], evaluating the antimicrobial activity of Wf, such metabolites as tannins, saponins, glycosides, and flavonoids were found to be responsible for the effect. However, in our study, the spectral data particularly revealed glycosides to be responsible for the antifungal effect. These findings are in accordance with those obtained by Dubey *et al*. [ 30 ], Grover *et al*. [ 31 ], and Kabesh *et al*. [ 32 ].

The antifungal activity of phytoglycosides has been mediated through multiple targets. The results of a study carried out by Zhang *et al*. [ 33 ] revealed that antifungal glycosides damage the plasma membrane, and that the leakage of cytoplasmic material causes cell death. Park *et al*. [ 34 ] indicated that this activity may be the result of the membrane disruption mechanisms. Likewise, Freiesleben *et al*. [ 35 ] suggested that after the complex formation of cholesterol, glycosides attach to the lyophilic moiety inside the membrane and hydrophilic moiety outside the cell, thereby suppressing the fungal growth.

A recently proposed mechanism by Lee et al. [ 36 ] is the inhibition of the calcineurin pathway. Calcineurin is a highly specific Ca^2+^-dependent serine- threonine phosphatase that has a key role in mediating stress cell response, which particularly helps in suppressing the growth of fungal species, primarily *C. albicans*. Accordingly, these possible mechanisms can account for the marked antifungal effect with the glycosides of Wfl as observed in our study. However, further research is required to determine the exact mechanisms involved in this particular effect.

The present study also involved the investigation of the antifungal effect of Pgp. Our results revealed a higher decrease in fungal colonies 1 week after the use of Pgp mouthwash. However, Wfl exhibited superior antifungal efficacy inducing a more significant decrease in viable colonies. There are no comparable studies addressing the antifungal activity of these two plants. Accordingly, the present study is the first of its kind, which effectively demonstrated the therapeutic protocol and determined the antifungal potential of these two plants.

The antifungal mechanism of Pgp has been confirmed by the presence of its active inhibitors, such as phenolics and flavonoids [ 37 ]. Different solvents have been used in various studies for the extraction of phytocompounds in different parts of *Punica*; however, it depends on the type of solvent used and the polarity. Lavaee *et al*. compared the efficacy of the methanolic and ethanolic extracts of the bark and roots of *Punica* in the isolation of *Candida* species and found that the methanolic extracts were more effective [ 38 ]. Accordingly, in the current study, ethanolic extracts were used.

Several studies have been performed on the antifungal effect of *Candida*. In this regard, Mansourian *et al*. reported that PPE had definite anti- Candidal activity based on the agar well diffusion method in vitro [ 39 ]. Likewise, Endo *et al*. described PPE as a potent inhibitor of *C. albicans* [ 40 ]. Moreover, Tayel *et al*. demonstrated that the application of PPE aerosol was an efficient method for complete sanitization and prevention against *C. albicans* growth in semi-closed places [ 41 ].

The presence of chemical components, such as flavonoids and terpenoids, in the current study revealed the antifungal effect of Pgp. Flavonoids inhibit fungal growth with several underlying mechanisms, such as the disruption of plasma membrane, induction of mitochondrial dysfunction, inhibition of the cell wall formation, cell division, RNA and protein synthesis, and efflux-mediated pumping systems [ 42 ]. Lolita *et al*. [ 43 ] evaluated the antifungal effect of the ethanolic extracts of red pomegranate peel against *C. albicans* and revealed flavonoids as the secondary metabolites in ethanolic extract responsible for the effect. The results of the mentioned study are evidently in accordance with our findings where the ethanolic extract of Pgp and metabolites, like flavonoids, were responsible for the antifungal effect.

Terpenoid was found to be another agent in Pgp accounting for the antifungal effect. The mechanism of action of this compound is the inhibition of morphogenesis, cell adhesion, and biofilm formation of *Candida* [ 44 ]. Based on the abovementioned findings, it is required to perform studies that can evaluate the in vivo use of phototherapeutic products, such as *W. fruticosa* and *Punica*, to obtain accurate results regarding their potential inhibitory effects against oral candidal infections and use them as a suitable target for discovering a novel anticandidal agent.

## Conclusion

The data obtained in our research indicated that Wfl was a potential inhibitor of oral *Candida* infection in patients with uncontrolled diabetes wearing removable dentures. The bioactive compounds, like glycosides, in Wf would have exerted their antifungal effect through multiple mechanisms, as described by various researchers. Furthermore, the advances in the discovery of new antifungal targets may help develop new formulations by encouraging the researchers to work on the isolation and characterization of phytoconstituents. Such new drug leads could help identify candidate medications for the treatment of the fungal infections of the oral cavity.
